# Sociodemographic factors associated with prenatal care utilization in Arkansas, United States

**DOI:** 10.1016/j.pmedr.2025.102983

**Published:** 2025-01-17

**Authors:** Pearl A. McElfish, Aaron Caldwell, Donya Watson, Jonathan Langner, Jennifer Callaghan-Koru, Austin Porter, Don E. Willis, Jennifer A. Andersen, Nicola L. Hawley, James P. Selig, Amir Forati, Maria R. Alcala, Lanita White, Enrique Gomez-Pomar, Clare C. Brown

**Affiliations:** a College of Medicine, University of Arkansas for Medical Sciences Northwest, Springdale, AR, USA; b Fay W. Boozman, College of Public Health, University of Arkansas for Medical Sciences Northwest, Springdale, AR, USA; c College of Medicine, University of Arkansas for Medical Sciences, Little Rock, AR, USA; d South Arkansas Regional Hospital, El Dorado, AR, USA; e Institute for Community Health Innovation, University of Arkansas for Medical Sciences Northwest, Springdale, AR, USA; f Arkansas Department of Health, Little Rock, AR, USA; g Department of Chronic Disease Epidemiology, Yale School of Public Health, New Haven, CT, USA; h Heartland Forward, Bentonville, AR, USA; i Community Health Centers of Arkansas, Little Rock, AR, USA; j Neonatology, St Bernards Regional Medical Center, Jonesboro, AR, USA; k Fay W Boozman College of Public Health, University of Arkansas for Medical Sciences, Little Rock, AR, USA

**Keywords:** Prenatal care, Maternal child health, Insurance payer, Race/ethnicity, Rural/urban residence

## Abstract

**Objectives:**

This study examines prenatal care utilization differences in Arkansas.

**Methods:**

Birth records data from the National Center for Health Statistics were used. The study population consisted of singleton live births in Arkansas from 2014 to 2022. Primary outcomes included number of prenatal visits, fewer than the recommended number of prenatal visits, late prenatal care, and no prenatal care. Summary statistics were computed, and adjusted rates ratios were calculated adjusting for maternal age, maternal education, and parity for payer, race/ethnicity, and rural/urban residence.

**Results:**

Mothers with a Medicaid-covered birth had 0.90 times fewer prenatal care visits and were 1.26 times more likely to have fewer than the recommended number of visits, 1.60 times more likely to initiate prenatal care late, and 1.46 times more likely to have reported no prenatal visits at all. Number of prenatal care visits was lower for all racial/ethnic groups relative to white mothers and for mothers living in rural areas. The largest differences were for Native Hawaiian and Pacific Islander (NHPI) mothers, who reported almost half the number of visits (ARR = 0.67, 95 % CI [0.64, 0.70]). Having no prenatal care was more prevalent among NHPI (ARR = 3.68, 95 % CI [2.66, 5.10]) and Black (ARR = 1.47, 95 % CI [1.34, 1.61]) mothers. Racially/ethnically minoritized groups were more likely to have late prenatal care and fewer than the recommended number of prenatal visits, with the greatest difference for NHPI.

**Conclusions:**

Findings document disparities in prenatal care utilization related to payer, race/ethnicity, and rural/urban residence.

## Highlights

• Mothers whose births were paid by Medicaid reported fewer prenatal care visits overall.
• Total number of prenatal care visits lower for all racial/ethnic groups relative to white mothers.
• Black and Native Hawaiian and Pacific Islander mothers more likely to have no prenatal care.
• Native Hawaiian and Pacific Islander mothers had almost half the visits as white mothers.
• Mothers in rural areas had lower prenatal care utilization.

## 1 Introduction

The United States (U.S.) has higher rates of maternal morbidity and mortality than other high-income nations (Tikkanen et al., 2022). Racially and ethnically minoritized women and women living in rural areas have higher rates of maternal morbidity and mortality compared to white women (Hoyert, 2024) and women living urban areas (Maron, 2017). For example, Black women have nearly three times the rates of severe maternal morbidity and maternal mortality than white women (Hoyert, 2024). Some studies suggest pregnant women of all races/ethnicities who live in rural areas have nearly double the maternal morbidity and mortality than women in large urban areas (Maron, 2017). Rural and racially and ethnically minoritized families also face disproportionately higher rates of infant mortality and preterm births (Kozhimannil et al., 2018; Laurore et al., 2020). Babies born in rural areas have a 20–40 % higher rate of preterm birth (Kozhimannil et al., 2018). Black infants experience 60 % higher rates of preterm birth and almost double the risk of infant mortality in their first year (MacDorman and Mathews, 2011). While there are many racial disparities related to maternal and infant mortality, it is important to acknowledge race as a social construct (Bryant et al., 2022). Births covered by Medicaid, of which there is a higher percentage in Southern states, have higher rates of severe maternal morbidity and mortality (Chen et al., 2021; Agency for Healthcare Research and Quality, 2024). However, little is known about the health care utilization patterns of mothers whose births are covered by Medicaid.

Arkansas, the location for this study, is a state with poor health rankings. Arkansas' infant mortality rate is considerably higher than the rest of the U.S (US Department of Health and Human Services HRSA, 2024), and Arkansas has one of the highest maternal mortality ratios of any state in the U.S (Centers for Disease Control and Prevention, 2024). Social determinants of health contribute to Arkansas' poor maternal outcomes—the state is largely rural (41 % of Arkansans vs 14 % U.S.), has higher poverty rates (16.8 % in Arkansas vs 12.6 % U.S.) (United States Census Bureau, 2024), and has a large percentage of births covered by Medicaid (41 %) (Kaiser Family Foundation, 2023). In recognition of these health maternal and infant health challenges in March of 2024, the governor of Arkansas signed an executive order to support mothers, protect babies, and improve maternal health (E.O. 24–03), with a specific call to better understand and improve utilization of prenatal care in Arkansas (Office of Governor Sarah Huckabee Sanders, 2024).

Initiating prenatal care early and maintaining regular appointments throughout pregnancy enhances a pregnant woman's health and the likelihood of delivering a healthy baby (American Academy of Pediatrics and American College of Obstetricians and Gynecologists, 2017). Early and adequate prenatal care has been associated with a substantial (43.8 %) decrease in postpartum maternal mortality (Liu et al., 2015), while receiving fewer than the recommended number of prenatal care visits is associated with higher rates of adverse maternal and infant outcomes (Partridge et al., 2012). Differential rates of prenatal care utilization may also contribute to racial/ethnic disparities in maternal outcomes. Previous studies have documented that Black women are more likely to receive inadequate prenatal care, which was associated with increased risk for maternal mortality and poor infant outcomes (Moaddab et al., 2016; Green, 2018). The American College of Obstetricians and Gynecologists recommend initiation of prenatal care as early as possible in pregnancy and ideally before the fourth month of gestation (American Academy of Pediatrics and American College of Obstetricians and Gynecologists, 2017). For most women, the number and recommended frequency of prenatal care visits is determined by the stage of pregnancy in which prenatal care was initiated. Following the recommended schedule, a woman with a low-risk pregnancy would receive 12 to 14 in-person prenatal care visits (Kotelchuck, 1997; Peahl and Howell, 2021). Adequate prenatal care is generally defined as initiating care within the first four months of gestation and receiving 80 % of the recommended number of prenatal visits (American Academy of Pediatrics and American College of Obstetricians and Gynecologists, 2017)

This study aims to examine associations between sociodemographic factors and prenatal care utilization among mothers in Arkansas, including total number of reported prenatal care visits, reporting fewer than the recommended number of prenatal care visits, reporting late initiation of prenatal care (at or after 4 months gestation), or reporting no prenatal care.

## 2 Methods

### 2.1 Data and population

Vital records data from the National Center for Health Statistics (NCHS) birth records were used. The study population consisted of singleton live births in Arkansas between January 1, 2014 and December 31, 2022. Given our goal to examine prenatal care utilization based on sociodemographic characteristics including insurance coverage, mothers were excluded from the current study if the delivery was self-pay, if the payer was listed as “other,” or if the payer was unknown. This study uses secondary data analysis and was deemed non-human subjects research by the University of Arkansas for Medical Sciences Institutional Review Board on September 12, 2023.

### 2.2 Variable definitions

The primary outcomes of interest included: 1) total number of reported prenatal care visits, 2) fewer than the recommended number of prenatal care visits, 3) late initiation of prenatal care, or 4) no prenatal care. Fewer than the recommended number of prenatal care visits was defined as receiving less than 80 % of the recommended number of prenatal care visits based on gestational age at birth (American Academy of Pediatrics and American College of Obstetricians and Gynecologists, 2017). Late initiation of prenatal care was defined as not receiving a prenatal care visit until after the third month of pregnancy (i.e., month 4 or later or not receiving any prenatal care). No prenatal care was defined as reporting zero prenatal care visits.

To assess differences in prenatal care utilization, we considered three key demographic variables of interest: payer, race/ethnicity, and rural/urban residence. Payer was defined as the payer for the birth and included Medicaid or private/other (i.e., private, TRICARE, or Indian Health Service [IHS]). We included IHS with private because there is less separate coverage compared to Medicaid. Maternal race/ethnicity was self-reported on the birth certificate and included non-Hispanic American Indian or Alaska Native (AIAN), Asian, Black, Hispanic (regardless of race), Native Hawaiian or Pacific Islander (NHPI), multiracial, and white. Rural-Urban Continuum codes from 2023 were used to designate the maternal county of residence as a metropolitan area (urban) or nonmetropolitan area (rural) using the classification provided by the U.S. Department of Agriculture (United States Department of Agriculture Economic Research Service, 2024).

Additional covariates included maternal information obtained from birth records. Specifically, we included maternal age (<20, 20–29, 30–39, ≥40 years); education (elementary [less than high school], secondary [graduated from high school], some college or higher); and live birth order (1, 2, or ≥ 3).

### 2.3 Statistical analyses

Summary statistics were computed for all study variables. Rates were calculated per 10,000 live births for the four study outcomes (i.e., total number of reported prenatal care visits, reporting fewer than the recommended number of prenatal care visits, reporting late initiation of prenatal care, and reporting no prenatal care). Adjusted rates ratios (ARRs) were calculated using estimated marginal means from multivariable modified Poisson regression models adjusting for maternal age, maternal education, and parity with a three-way interaction for payer, race/ethnicity, and rural/urban residence. The interaction was included because of the possible effects of interconnection of these variables (e.g., the effect of rural/urban residence may be moderated by race and/or payer). Additionally, gestational age was also included as a covariate in the number of prenatal care visits model since those with a higher gestational age would have greater opportunity for more prenatal care visits. We used the “emmeans” R package with CIs calculated using an asymptotic method with a multivariate-t adjustment for multiple comparisons (Searle et al., 1980). Statistical significance was assumed at *p* < 0.05. All statistical analyses were performed using R version 4.3.2.

## 3 Results

### 3.1 Descriptive statistics

We identified 333,460 singleton live births in Arkansas, of whom 298,055 indicated Medicaid or private insurance (including TRICARE and IHS) as the primary payer. Characteristics of the study population can be seen in Table 1. Among the sample, the average number of reported prenatal care visits was 10 (SD = 4), 43.0 % reported fewer than the recommended number of prenatal care visits, 29.0 % reported late initiation of prenatal care, and 2.3 % reported no prenatal care.

**Table 1:** Characteristics of study population among women who gave birth in Arkansas (2014–2022).

Characteristic	N	%
Mother's Race	296,917	
AIAN		0.5 %
Asian		2.2 %
Black		19 %
Hispanic		11 %
Multiracial		1.7 %
NHPI		1.0 %
White		65 %
Urban or Rural County of Residence	298,055	
Rural		39 %
Urban		61 %
Payer	298,055	
Medicaid		47 %
Private		53 %
Mother's Age	298,055	
< 20 years		8.5 %
20–29 years		60 %
30–39 years		30 %
≥ 40 years		1.7 %
Mother's Education Level	295,946	
Elementary		2.2 %
Secondary		44 %
Some post-secondary or college		53 %
Parity	296,072	
1		33 %
2		27 %
3 or more		40 %

Note: AIAN = American Indian or Alaska Native; NHPI=Native Hawaiian or Pacific Islander.

**Fig. 1:**
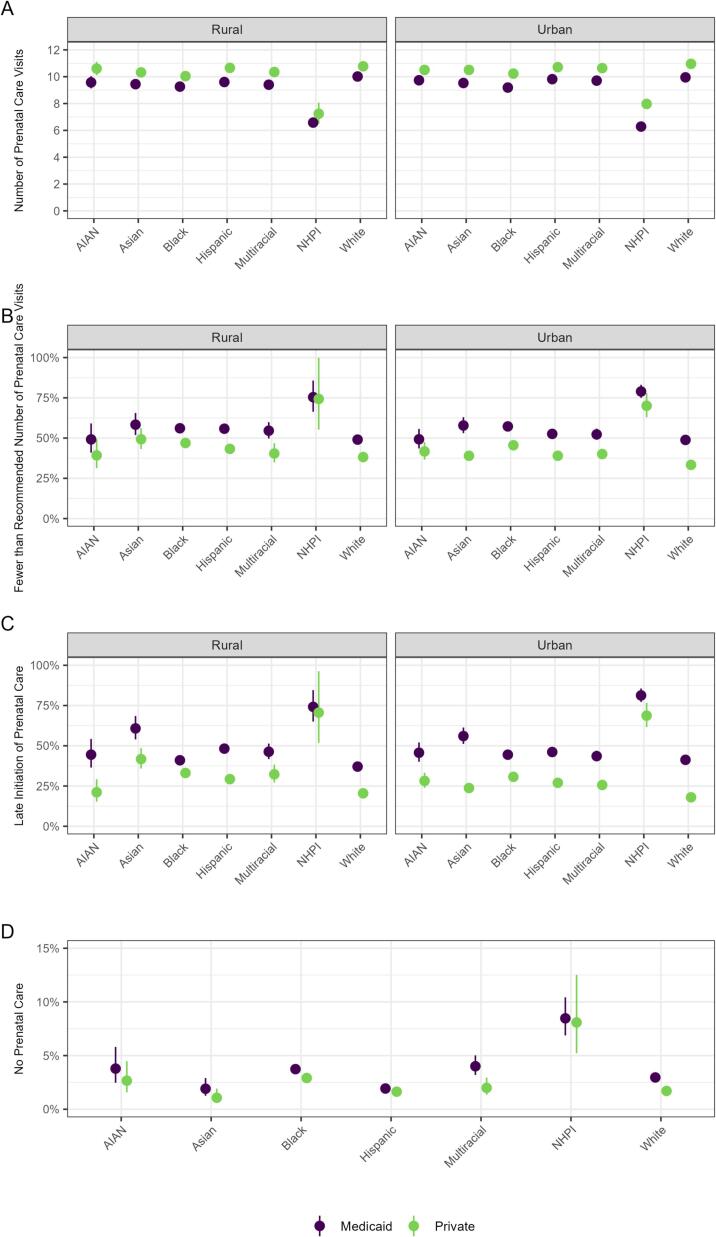
Prenatal care utilization among women who gave birth in Arkansas (2014–2022) separated by race/ethnicity, rurality, and payer. Caption: Point estimates and 95 % CIs for modified Poisson model marginal means (adjusted for age, education, and parity; in panel A gestational age is also a covariate in the model) are displayed for A) predicted total number of prenatal care visits, B) predicted probability of fewer than the recommended number of prenatal care visits, C) predicted probability of late initiation of prenatal care, and D) predicted probability of no prenatal care. Please note that rurality is not provided for panel D due to cell sizes <10. Note: AIAN = American Indian or Alaska Native; NHPI=Native Hawaiian or Pacific Islander.

Fig. 1 displays the average number of prenatal care visits and adjusted predicted probability of fewer than recommended number of prenatal care visits, late initiation of prenatal care, and no prenatal care by payer, race/ethnicity, and rural/urban residence.

### 3.2 Differences by payer

Those receiving Medicaid (adjusting for maternal age, parity, education, and race/ethnicity) had lower/worse prenatal care utilization across all outcomes (Table 2). Mothers with a Medicaid-covered birth had 0.90 times fewer prenatal care visits, were 1.26 times more likely to have fewer than the recommended number of visits, were 1.60 times more likely to initiate prenatal care late, and were 1.46 times more likely to have reported no prenatal visits at all.

**Table 2:** Marginal effects of Medicaid on prenatal care utilization among women who gave birth in Arkansas (2014–2022).

Outcome	ARR	95 % C.I.		
Number of Prenatal Care Visits^a^	0.90	[0.89, 0.91]		
Fewer than Recommended Number of Prenatal Care Visits^b^	1.26	[1.21, 1.31]		
Late Initiation of Prenatal Care^b^	1.60	[1.53, 1.68]		
No Prenatal Care^b^	1.46	[1.23, 1.73]		

Note: Private as the reference group (Medicaid/Private); ARR = adjusted rate ratio; CI = confidence interval.

^a^Adjusted for gestational age, urban/rural residence, payer, maternal age, education, and parity.

^b^Adjusted for urban/rural residence, payer, maternal age, education, and parity.

### 3.3 Differences by race/ethnicity

Prenatal care utilization differed across race/ethnicity (Table 3). The number of prenatal care visits was lower for all racial/ethnic groups relative to white mothers, with the largest differences found for NHPI mothers.

**Table 3:** Marginal effect of race/ethnicity on prenatal care utilization among women who gave birth in Arkansas (2014–2022).

	AIAN	Asian	Black	Hispanic	Multiracial	NHPI
Number of Prenatal Care Visits^a^						
ARR	0.97	0.95	0.93	0.98	0.96	0.67
95 % C.I.	[0.94, 1.00]	[0.94, 0.97]	[0.92, 0.93]	[0.97, 0.98]	[0.95, 0.98]	[0.64, 0.70]
						
Fewer than Recommended Number of Prenatal Care Visits^b^						
ARR	1.07	1.21	1.22	1.13	1.11	1.78
95 % C.I.	[0.95, 1.20]	[1.13, 1.29]	[1.20, 1.25]	[1.10, 1.16]	[1.04, 1.19]	[1.59, 2.00]
						
Late Initiation of Prenatal Care^b^						
ARR	1.21	1.56	1.35	1.33	1.31	2.68
95 % C.I.	[1.05, 1.40]	[1.45, 1.69]	[1.31, 1.38]	[1.28, 1.38]	[1.21, 1.42]	[2.38, 3.03]
						
No Prenatal Care^b^						
ARR	1.41	0.64	1.47	0.79	1.26	3.68
95 % C.I.	[0.90, 2.22]	[0.39, 1.03]	[1.34, 1.61]	[0.68, 0.92]	[0.93, 1.70]	[2.66, 5.10]

Note: White as the reference group; AIAN = American Indian or Alaska Native; NHPI=Native Hawaiian or Pacific Islander.

^a^Adjusted for gestational age, urban/rural residence, payer, maternal age, education, and parity.

^b^Adjusted for urban/rural residence, payer, maternal age, education, and parity.

Having fewer than the recommended number of prenatal care visits was significantly more prevalent in all racial/ethnic groups compared to white mothers, except for AIAN mothers.

All racial/ethnic groups were more likely to have late initiation of prenatal care compared to white mothers. The greatest difference was again for NHPI mothers.

Having no prenatal care was more prevalent among NHPI (ARR = 3.68, 95 % CI [2.66, 5.10]) and Black (ARR = 1.47, 95 % CI [1.34, 1.61]) mothers relative to white mothers. However, Hispanic (ARR = 0.79, 95 % CI [0.68, 0.92]) mothers were less likely to have reported no prenatal care visits. There were no significant differences between white mothers and Asian, AIAN, or multiracial mothers.

### 3.4 Differences by rural/urban residence

Those in rural areas were more likely to report late initiation of prenatal care (ARR = 1.05, 95 % CI [1.00, 1.10]) and reported fewer prenatal care visits (ARR = 0.99, 95 % CI [0.97, 1.00]; Table 4). After adjusting for covariates, there were no significant differences in having no prenatal care utilization or having fewer than the recommended number of prenatal care visits between individuals living in urban and rural areas.

**Table 4:** Marginal effect of Rural-Urban Continuum on prenatal care utilization among women who gave birth in Arkansas (2014–2022).

Outcome	ARR	95 % C.I.		
Number of Prenatal Care Visits^a^	0.99	[0.97, 1.00]		
Fewer than Recommended Number of Prenatal Care Visits^b^	1.04	[1.00, 1.08]		
Late Initiation of Prenatal Care^b^	1.05	[1.00, 1.10]		
No Prenatal Care^b^	0.89	[0.75, 1.06]		

Note: Urban as the reference group (Rural/Urban); ARR = adjusted rate ratio; CI = confidence interval.

^a^Adjusted for gestational age, urban/rural residence, payer, maternal age, education, and parity.

^b^Adjusted for urban/rural residence, payer, maternal age, education, and parity.

## 4 Discussion

Early and adequate prenatal care utilization has consistently been demonstrated to improve health outcomes for both mothers and babies (Partridge et al., 2012; Moaddab et al., 2016). This study used Arkansas birth records to examine if sociodemographic (payer, race/ethnicity, rural/urban residence) factors were associated with prenatal care utilization. Mothers whose births were paid by Medicaid reported fewer prenatal care visits overall, were more likely to have fewer than the recommended number of prenatal care visits, were less likely to have early prenatal care, and were more likely to have no prenatal care at all. Pregnant women in Arkansas are eligible for Medicaid if their household income is at or below 214 % of the federal poverty limit (Healthinsurance.org, 2024). Although this study does not examine why Medicaid was associated with significantly less prenatal care utilization, several policies and practices may contribute. Arkansas did not have presumptive Medicaid during the period analyzed, which means women must wait until their Medicaid application is complete and approved to utilize prenatal care. Many health care providers will not see a patient until their Medicaid is approved (Bellerose et al., 2022). Some studies have shown an increase in early prenatal care after implementing presumptive eligibility (Eliason and Daw, 2022). Furthermore, many health care providers limit the number of Medicaid patients they will take, and patients may have to make several attempts to find a provider and schedule an appointment (Bellerose et al., 2022). In addition, during the study period, Arkansas Medicaid utilized a bundled payment approach for perinatal care, reducing financial incentive for more prenatal care visits or for seeing women early in their pregnancy (Arkansas Perinatal Episode of Care, 2021). Because Medicaid eligibility is based on income, individuals on Medicaid have lower household incomes, and they may face other barriers to prenatal care utilization such as lower health literacy and challenges with transportation and childcare (Bellerose et al., 2022).

Prenatal care utilization also varied based on race/ethnicity. The total number of prenatal care visits was lower for all racial/ethnic groups relative to white women. The largest differences were found for NHPI mothers, who reported almost half the number of visits compared to white mothers. Both Black and NHPI mothers were more likely to have no prenatal care at all, with NHPI mothers being four times more likely to receive no prenatal care. Interestingly, Hispanic mothers were less likely to have no prenatal care comparted to white women. All racially and ethnically minoritized groups were more likely to have late initiation of prenatal care, and all racially and ethnically minoritized groups (except for AIAN) were more likely to have fewer than the recommended number of prenatal care visits compared to white mothers, with the greatest difference between NHPI and white mothers. These findings are consistent with the limited literature examining factors associated with prenatal care utilization which have documented disparities among Black and Hispanic populations (Moaddab et al., 2016; Green, 2018).

This article provides new information documenting disparities in prenatal care utilization among AIAN, Asian, multiracial, and NHPI mothers, who have largely been left out of the literature on maternal health. Many NHPI in Arkansas are Compact of Free Association Migrants from the Republic of the Marshall Islands. Prior qualitative research with Marshallese Pacific Islander mothers has identified several barriers to prenatal care including difficulty understanding and navigating Medicaid, a lack of transportation to get to appointments, and language barriers (Ayers et al., 2018). Prior research with Marshallese mothers also identified a lack of understanding of the importance of early and consistent prenatal care as well as experiences with discrimination and fear when seeking prenatal care (Ayers et al., 2018).

Our findings that Black mothers completed fewer prenatal care visits, regardless of insurance coverage, is concerning given that prior studies have documented higher rates of comorbidities among Black maternity patients (Brown et al., 2020). While the typical prenatal visit schedule includes 12 to 14 visits, the current *Guidelines for Perinatal Care* recommend that the frequency of prenatal care visits be individualized based on each patient's risks and needs (American Academy of Pediatrics and American College of Obstetricians and Gynecologists, 2017). Mothers with comorbidities require more frequent visits (American Academy of Pediatrics and American College of Obstetricians and Gynecologists, 2017).

Racially and ethnically minoritized mothers experience higher rates of maternal morbidity and mortality (Hoyert, 2024), and their children have higher rates of preterm birth, low birthweight, and infant death (MacDorman and Mathews, 2011). Early and adequate prenatal care has consistently been demonstrated to reduce negative outcomes for both mothers and babies (Partridge et al., 2012; Moaddab et al., 2016). Therefore, it is imperative to address racial disparities in prenatal care utilization.

There was a small but statistically significant difference in prenatal care utilization between mothers living in rural and urban areas. Rural women have less access to obstetrical services (Maron, 2017), and rural populations generally have farther distances to travel when seeking prenatal care. This is particularly true in counties with higher proportions of minoritized women and lower median household incomes (Hung et al., 2017). These factors may all contribute to the differences in prenatal care utilization.

### 4.1 Recommendations for policy and practice

Arkansas currently has among the worst maternal and infant outcomes in the U.S (US Department of Health and Human Services HRSA, 2024; Centers for Disease Control and Prevention, 2024). Early and adequate prenatal care is associated with better maternal and infant outcomes (Liu et al., 2015; Partridge et al., 2012; Moaddab et al., 2016). This research can inform health care providers and policy makers as Arkansas seeks to improve maternal and infant health outcomes by improving the utilization of prenatal care. Several policy changes have the potential to improve these outcomes. First, Arkansas should consider implementing presumptive eligibility for pregnancy Medicaid, which allows mothers to have temporary eligibility while their application is being processed. States that implemented presumptive eligibility have shown an increase in early prenatal care (Eliason and Daw, 2022). Second, Arkansas should consider expanding the use of and reimbursement for community health workers and doulas. Community health workers and doulas have demonstrated the ability to improve prenatal care utilization by helping mothers overcome socio-economic barriers such as transportation, language, low-health literacy, and fear or uncertainty when navigating the health care system (McCue et al., 2022). Community health workers and doulas have also helped mitigate racial/ethnic inequalities within the health system (Moore et al., 2020). Several states provide some Medicaid coverage for community health workers and community-based doulas (Institute for Medicaid Innovation, 2023). Third, Arkansas could also consider changes to the way Medicaid reimburses for prenatal care. Currently, Arkansas uses global bundled payment which pays at delivery. Arkansas could consider performance-based incentives for providers to provide the recommended number of visits or meet other quality measures. Fourth, there also may be opportunities to better leverage the mobile prenatal care and local health units to reduce transportation burdens, particularly in rural areas, which may improve early prenatal care and increase the number of prenatal care visits (Edgerley et al., 2007). Fifth, Arkansas could also consider the promotion of group prenatal care programs such as CenteringPregnancy, which has been demonstrated to increase the amount of prenatal education and care a mother receives and has demonstrated health benefits for mothers and infants, especially among Black mothers (Crockett et al., 2022). Sixth, targeted, culturally-appropriate prenatal care outreach and educational programs may be needed to reach NHPI community members who have the lowest prenatal care utilization. While the study was conducted in Arkansas, these recommendations are relevant for other states with large Medicaid populations and those serving NHPI communities.

### 4.2 Limitations

Findings from this study should be interpreted with some limitations. First, we utilized data from birth records in the state of Arkansas, and our results may not be generalizable to other states. Second, we have examined associations; our results do not establish causality. Third, some questions remain regarding the reliability and validity of birth certificate data (Martin et al., 2013). Fourth, our assessment of timing and quantity of prenatal care does not tell us about the content or quality of the care. Fifth, we could not adjust for a mother's risk level and the number of visits needed, and therefore, we cannot speak to whether utilization was “adequate” for high-risk women. These limitations may create potential biases. However, even with these limitations, the results yield valuable insights into prenatal care utilization and the potential risk of mothers who live in rural areas, are covered by Medicaid, and/or are from specific racial/ethnic groups. The study is also strengthened by the large sample size.

## Funding

Research reported in this publication was supported by 10.13039/100008519University of Arkansas for Medical Sciences Translational Research Institute funding awarded through the 10.13039/100006108National Center for Advancing Translational Sciences of the 10.13039/100000002National Institutes of Health (UL1 TR003107). This work was also supported by the Community Engagement Alliance (CEAL) Against COVID-19 Disparities (NIH 10T2HL156812–01) and by a Racial and Ethnic Approaches to Community Health (REACH) award (6 NU58DP007601–01-01) from the 10.13039/100000030Centers for Disease Control and Prevention. CCB is supported by the National Institute on Minority Health and Health Disparities (NIMHD) of the National Institutes of Health (NIH; 1K01MD018072). The content is solely the responsibility of the authors and does not necessarily represent the official views of the funders.

## CRediT authorship contribution statement

**Pearl A. McElfish:** Writing – review & editing, Writing – original draft, Resources, Project administration, Investigation, Funding acquisition, Conceptualization. **Aaron Caldwell:** Writing – review & editing, Formal analysis, Data curation, Conceptualization. **Donya Watson:** Writing – review & editing, Conceptualization. **Jonathan Langner:** Writing – review & editing, Conceptualization. **Jennifer Callaghan-Koru:** Writing – review & editing, Conceptualization. **Austin Porter:** Writing – review & editing, Formal analysis, Data curation, Conceptualization. **Don E. Willis:** Writing – review & editing, Conceptualization. **Jennifer A. Andersen:** Writing – review & editing, Conceptualization. **Nicola L. Hawley:** Writing – review & editing, Formal analysis, Data curation, Conceptualization. **James P. Selig:** Writing – review & editing, Formal analysis, Data curation, Conceptualization. **Amir Forati:** Writing – review & editing, Conceptualization. **Maria R. Alcala:** Writing – review & editing, Conceptualization. **Lanita White:** Writing – review & editing, Conceptualization. **Enrique Gomez-Pomar:** Writing – review & editing, Conceptualization. **Clare C. Brown:** Writing – review & editing, Formal analysis, Data curation, Conceptualization.

## Declaration of competing interest

The authors declare that they have no known competing financial interests or personal relationships that could have appeared to influence the work reported in this paper.

## Data Availability

Vital records data from the National Center for Health Statistics (NCHS) birth records were used.
